# Thymoquinone combined with conventional antibiotics against pandrug-resistant *Staphylococcus aureus*: a pharmacodynamic and molecular simulation strategy to overcome bioavailability limitations

**DOI:** 10.3389/fphar.2026.1735325

**Published:** 2026-03-12

**Authors:** Adel Attia M. Ahmad, El-Sayed Y. M. El-Naenaeey, Gamal A. Elmowalid, Tarek Khamis, Touka A. Kandel, Dina M. Khodeer, Razan Orfali, Marwa I. Abd El-Hamid

**Affiliations:** 1 Department of Microbiology, Faculty of Veterinary Medicine, Zagazig University, Zagazig, Egypt; 2 Department of Pharmacology, Faculty of Veterinary Medicine, Zagazig University, Zagazig, Egypt; 3 Department of Pharmacology, College of Medicine, Imam Mohammad Ibn Saud Islamic University (IMSIU), Riyadh, Saudi Arabia

**Keywords:** anti-efflux activity, cell wall-related genes, Nigella sativa, *Staphylococcus aureus*, thymoquinone

## Abstract

**Background:**

Infections with pandrug-resistant (PDR) *Staphylococcus aureus* (*S. aureus*) present no available treatment alternatives, even when employing most antibacterial combination regimen. Restoring the effectiveness of existing antibiotics especially through the integration of safe plant-derived compounds represents a promising therapeutic approach. Despite extensive *in vitro* evidence of thymoquinone’s (TQ, *Nigella sativa* extract) bactericidal activity against *S. aureus*, its clinical application remains limited due to extremely low serum maximum concentration (Cmax), which is far below the minimal inhibitory concentrations (MICs) required for most clinical isolates. This study aimed to overcome this pharmacokinetic barrier by identifying synergistic antibacterial combinations that enhance TQ efficacy at clinically achievable concentrations.

**Methods:**

Pharmacodynamic interactions between TQ or *Nigella sativa* essential oil and selected antibiotics were evaluated in treated *Staphylococcus aureus* strains using disc replacement and modified checkerboard assays. To investigate the molecular response, the expression levels of *norA*, *PBP2a* and *PBP4* genes were quantified through real-time PCR analysis. Furthermore, molecular dynamics simulations were performed, for the first time, to demonstrate TQ’s direct inhibitory action on PBP2a and PBP4 proteins.

**Results:**

Both TQ and individual antibacterials initially exhibited MIC values consistent with bacterial resistance. However, when TQ was combined with ciprofloxacin and one of azithromycin, clindamycin, gentamicin or amoxicillin/clavulanic acid, a marked pharma-codynamic enhancement was observed leading to 8 to 10-fold reductions in TQ-MICs. These combinations successfully restored antibacterial activity as evidenced by fractional inhibitory concentration index values ranging from 0.0002 to 0.01. Additionally, TQ contributed to the prevention of antagonistic interactions between certain dual antibacterial pairings. The combination interactions were closely associated with downregulation of *norA, PBP2a* and *PBP4* genes as well as the direct inhibition of PBP2a and PBP4 proteins through thymoquinone ligands.

**Conclusion:**

Pharmacodynamic interactions in *S. aureus* treated with certain dual antibacterial combinations alongside TQ led to a significant reduction in MIC values, downregulated *norA, PBP2a* and *PBP4* genes and directly inhibited PBP2a and PBP4 proteins through TQ ligand binding. These findings provide new perspectives on enhancing the clinical applicability of TQ reviving the efficacy of conventional dual antibacterial therapies.

## Introduction

*Staphylococcus aureus* (*S. aureus*) is a highly adaptable human pathogen that causes a wide spectrum of hospital- and community-acquired infections, largely due to its extensive repertoire of virulence factors (Ammar et al., 2016). In addition to human disease, *S. aureus* is a major cause of intramammary infections in cattle and represents the primary etiological agent of contagious clinical and subclinical mastitis in dairy herds (Abd El-Hamid et al., 2024). The pathogenic potential of this bacterium is driven by a complex and tightly regulated process that involves the coordinated expression of numerous virulence determinants at different stages of infection, controlled by an intricate regulatory network. These determinants include a wide range of exoproteins as well as proteins associated with the bacterial cell wall. Almost all *S. aureus* strains produce a set of secreted exoproteins such as exotoxins and enzymes including coagulase, nucleases, proteases, lipases, hyaluronidase and collagenase (Ahmad et al., 2025; Abd El-Hamid et al., 2019). Antibacterial-drug-resistant *Staphylococcus aureus* is a major public health concern and threatens medical advancements relying on the availability of successful antibacterial agents. In the future, the absence of interventions to reduce the development of antibacterial resistance will lead to global morbidity and mortality. During the last COVID-19 epidemics, a 54.5% increase in methicillin resistant *S. aureus* (MRSA) infection and colonization was reported (Abubakar et al., 2023). *S. aureus* small-colony variants (SCVs) exhibit slow growth, metabolic deficiencies and strong persistence with diagnostic and therapeutic challenges (Ye et al., 2025). They in chronic wounds are frequently under-detected and alter antimicrobial susceptibility underscoring the urgent need for improved diagnostic approaches (Carris et al., 2025). During the treatment course of *S. aureus* infections, it evolves various mechanisms of resistance to antibiotics (Mwangi et al., 2007; Snitser et al., 2020) and shifts to an untreated subpopulation of bacterial persisters (Peyrusson et al., 2020; Garzoni and Kelley, 2011). Therefore, medical practitioners are used to depend on multiple antibiotics to overcome these infections (Tyers and Wright, 2019). Antibacterial activity of synthetic or natural products is estimated by disc diffusion assays, broth microdilution, resazurin assay, checkerboard assay, time-kill kinetics, virtual screening (docking) and gene regulation (Sarker et al., 2007; Adusei et al., 2019; Depardieu et al., 2007; Black et al., 2023). Synergistic antibiotics are advised for rapid and efficient clearance of infections, reduced dose-related toxicity, total bacterial eradication and efficient bactericidal effects against the evolutionary selection of resistant strains (Acar, 2000; Leekha et al., 2011). Synergistic antibiotic combinations of tobramycin/vancomycin, daptomycin/tobra-mycin, ciprofloxacin/daptomycin and ciprofloxacin/vancomycin produced bactericidal effects against *S. aureus* biofilm with a reduced minimum inhibitory concentration (MIC) (Kamble et al., 2023). The combinations of fluoroquinolones with oxacillin were synergistic against MRSA, but not methicillin sensitive *S. aureus* (MSSA) isolates (Rohner et al., 1989). The synergy of ciprofloxacin with oxacillin, gentamycin or vancomycin involved only 50% of examined *S. aureus* strains, while others recoded indifference (Bauernfeind and Petermuller, 1984). Fluoroquinolones produced infrequent synergy with amino-glycosides, β-lactams, clindamycin, macrolides and imidazoles among Gram-positive bacteria (Neu, 1991). There was no agreement between the *in vitro* antagonism of rifampicin and ciprofloxacin and that observed in experimental animals (Kaatz et al., 1989). Many agents combined with ciprofloxacin revealed addition and some combinations of ciprofloxacin with one of the following: doxycycline, erythromycin, clindamycin or chloramphenicol showed antagonistic interactions against some MSSA (Hailer, 1987). Controversy exists over ciprofloxacin synergy with several antibacterials in some strains and indifference for the rest (Hailer, 1987; Chin et al., 1986). Thymoquinone MICs against *S. aureus* strains were widely variable in several investigations; 50 μg/mL (Goel and Mishra, 2018), 53.3–106.6 μg/mL (Rondevaldo et al., 2015), 8–32 μg/mL (Chaieb et al., 2011) and 8–256 μg/mL (Kouidhi et al., 2011). These levels were extremely higher than TQ-blood concentration maximum (Alkharfy et al., 2011; Elmowafy et al., 2016). Restoration of antibacterials activity by *Nigella sativa* (*N. sativa*) essential oil or TQ was explored; supplementation of *N. sativa* essential oil at 0.5XMIC induced a 4- to 8-fold decrease in the minimum inhibitory concentration (MIC) of ciprofloxacin against *S. aureus* strains (Mouwakeh et al., 2019). Similarly, supplementation of TQ at 0.5XMIC induced a 4- to 8-fold decrease in the MIC of tetracyclines against *S. aureus* strains (Kouidhi et al., 2011). The combined activity of TQ with oxacillin, penicillin and tetracyclines showed 0.263 to 0.450, 0.466, and 0.400 to 0.475 fractional inhibitory concentration (ΣFIC), respectively against *S. aureus* strains (Rondevaldo et al., 2015). Thymoquinone inhibits the antibacterial efflux, prevents the pump of ethidium bromide out of *S. aureus* as described in the ethidium bromide accumulation assay and reduces the expression of *mepA* efflux gene (Mouwakeh et al., 2019). The efflux pump inhibitor drug pyrvinium, combined with ciprofloxacin, produced remarkable potency *in vitro* against *S. aureus* biofilm (Mahey et al., 2021). TQ exerts direct effects on *S. aureus* cell envelops interfering with enzymes of cell wall synthesis, cell wall fibrillar detachment, cytoplasmic vacuolation and disorganization of the mitochondrial and nuclear structures of *Aspergillus fumigatus* and *Aspergillus flavus* (Khosravi et al., 2011). Similar effects on *Candida* species included cytoplasmic membrane separation from the thickened cell wall and cytoplasmic disorganization (İşcana et al., 2016). Sub-inhibitory concentrations of *N. sativa* essential oil were accompanied by downregulation of *PBP2a* and *murF* genes in treated *S. aureus* strains (Attia et al., 2016). Penicillin-binding proteins (PBPs) catalyze the enzymes that create the peptidoglycan assembly in the bacterial cell wall. PBP2a is encoded by MRSA and is responsible for the resistant phenotype. The low affinity of PBP2a for transpeptidase-inhibiting β-lactams allows MRSA to continue cell wall assembly synthesis in the presence of β-lactams (Fergestad et al., 2020). PBP4 (pbpD) is a dimeric muropeptide cross-linked with pentaglycine bridges in the cell wall and inhibition of pbpD affects ceftizoxime resistance (Łeski and Tomasz, 2005). From the abovementioned issues, the aim of this study was to address the clinical limitations of TQ by investigating strategies that enable its bactericidal activity against *S. aureus* at concentrations achievable within the serum C-max range.

## Materials and methods

### Thymoquinone and *Nigella sativa* essential oil

The study utilized thymoquinone (TQ, 274666-5G, San Louis, MO, United States) and pure cold pressed extracted (20.0%) *Nigella sativa* (NS) essential oil (Harraz Herbal Products Company, Egypt). *Nigella sativa* essential oil, often called black cumin seed oil, was extracted from the black seeds of NS plant and it is rich in thymoquinone Stock preparations were made by separately dissolving 250 mg of thymoquinone and 10 mL of NS oil in 10% DMSO (San Louis, MO, United States). These solutions were kept frozen at −20 °C and working concentrations were obtained by diluting 1 mL of stock solutions with equal parts of ethanol and water immediately before use.

### *Staphylococcus aureus* isolation and characterization

A total of 50 samples were obtained from three different sources to isolate *S. aureus* strains; human clinical specimens, food market products and dairy farm materials. The human samples included turbid urine (no = 20), pus from diabetic foot ulcers (no = 3) and pleural fluid (no = 1), all sourced from private microbiology laboratories in Zagazig city, Sharkia Governorate, Egypt. Animal samples encompassed food market ones; minced meat (no = 6) and luncheon (no = 5) and milk from mastitis affected cows (no = 15). Sample collection was performed aseptically in November 2023 with immediate delivery to the Bacteriology Laboratory Unit, Faculty of Veterinary Medicine, Zagazig University. A loopful from each aseptically prepared sample was inoculated into mannitol salt broth (Oxoid, United Kingdom) and then the tubes were incubated at 37 °C for 24 h. A loopful of bacterial growth was inoculated onto mannitol salt agar plates, which were incubated at 37 °C for 24 h. The resulting colonies were assessed for beta hemolytic activity on sheep blood agar. Typical colonies that emerged were analyzed for free coagulase production using established procedures (Barry et al., 1973). Protein A detection in the growing colonies was performed using the Staphytect Plus latex slide agglutination test kits (ThermoFisher, Code: DR0850) as per manufacturer’s protocol. Isolates that tested positive for both coagulase and protein A were stored in glycerol at −80 °C.

### Disc diffusion assays

Susceptibility testing of the recovered *S. aureus* isolates was evaluated using 15 commercial antibacterial discs (Oxoid, United Kingdom). The antibacterial effects of NS oil and TQ were determined using 6 mm Whatman filter paper discs No. 1 saturated with NS oil (20 µL dissolved in 10% DMSO/disc, which was equivalent to 20 mg of *N. sativa* seeds) or TQ (20 μL, 50 µg/disc) according to the previously described methods (Jorgensen and Turnidge, 2015). For susceptibility testing, bacterial suspensions at 10^8^ CFU/mL in phosphate buffer saline (PBS) were plated onto Mueller-Hinton agar (MHA) plates. Commercial antibiotic discs including ampicillin (AMP), ampicillin/sulbactam (SAM), amoxicillin/clavulanic acid (AMC), trimethoprime/sulpha-methoxazole (SXT), ciprofloxacin (CIP), doxycycline (DO), linezolid (LNZ), azithromycin (AZM), fosfomycin (FF), oxacillin (OX), clindamycin (DA), gentamicin (CN), cefotaxime (CTX), chloramphenicol (C) and ceftazidime (CZ) were placed on the dried MHA plates. For NS oil and TQ susceptibility testing, the prepared discs were placed on the inoculated dried MHA plates. Following incubation at 37 °C for 24 h, inhibition zone diameters were measured and recorded. The multiple antibiotic resistance (MAR) index for each isolate was estimated utilizing the formula; MAR = a/b, where a is the number of antibacterial agents to which the isolates were resistant and b is the total number of antibacterial agents tested (Abdel-Raheem et al., 2023).

### Co-application of *N. sativa* essential oil or thymoquinone with antibacterial compounds on Mueller-Hinton agar plates

The PDR *S. aureus* strains that showed resistance to NS essential oil and TQ and also exhibited resistance to a combination of antibacterial therapies were then tested for the adjuvant effects of NS oil or TQ as described below.

### Disc replacement assay for assessing combined effects of *N. sativa* essential oil and individual antibacterials

Disc replacement technique determining whether NS essential oil enhanced the antibacterial activities of trimethoprime/sulphamethoxazole, ciprofloxacin, doxycyclins, linezolid, azithro-mycin, fosfomycin, oxacillin, clindamycin, gentamycin, cefotaxime or cephazoline against *S. aureus* resistant strains was utilized using an established protocol (Al Laham and Al Fadel, 2014). The procedure involved preparing a bacterial suspension of PDR *S. aureus* strains at a concentration of 10^8^ cells/mL of PBS, which was then spread onto MHA agar plates. Filter paper discs (Whatman No. 1) were saturated with 20 µL of NS essential oil and positioned on the agar surface. After an incubation period of 15 min at - 4 °C, the oil-treated discs were removed and replaced with standard antibiotic discs for the examined individual antibacterials at the same locations. The plates underwent incubation at 37 °C for 24 h, after which the diameters of the zones of growth inhibition were measured to assess the synergistic individual antibacterial effects.

### Disc replacement assay for assessing combined effects of *N. sativa* essential oil or thymoquinone and two individual antibacterials

Disc replacement technique determining whether NS essential oil or TQ could potentiate the individual antibacterial efficacy of two combined antibacterials against PDR *S. aureus* strains was used following a standardized methodology (Soltani et al., 2012). The experimental approach involved creating bacterial inocula from PDR *S. aureus* strains at a concentration of 10^8^ CFU/mL in PBS, which were then plated on MHA plates. Filter paper discs (Whatman No. 1) were impregnated with 20 μL of NS essential oil and placed on the agar surface. Following a pre-incubation period of 15 min at – 4 °C, the oil-containing discs were removed and substituted with the two standard antibacterial discs at the same positions. The plates were then incubated at 37 °C for 24 h and inhibition zone diameters were measured to evaluate synergistic antibacterial interactions between the two antibacterials in the vicinity of NS on MHA plates. An analogous procedure was performed using TQ-saturated discs (20 μL, 50 μg per disc). Antibacterials’ pairs lacking NS oil or TQ treatment served as controls. The antibacterial combinations exhibiting synergistic interactions on solid media were chosen for subsequent testing.

### Minimum inhibitory concentration determination

The individual or paired antibacterial agents that demonstrated synergistic activity with TQ on MHA agar were subjected to MICs determination against *S. aureus* isolates according to CLSI guidelines (Clinical and Laboratory Standards Institute CLSI, 2020). Briefly, serial two-fold dilutions of 100 μL of TQ or antibacterials in Mueller-Hinton broth were dispensed in microtiter plates. An equal volume of Mueller-Hinton broth containing fresh *S. aureus* bacterial inoculum (10^5^ CFU/mL) was added to each well. The sealed plates were incubated at 37 °C for 24 h. Triplicate testing was conducted and the mean MIC values demonstrating total bacterial growth suppression were recorded. The resulting MIC data were documented to define the parameters for the modified checkerboard analysis.

### Modified checkerboard analysis for assessing combined effects of thymoquinone and two combined antibacterials against resistant *S. aureus*

Three-dimensional checkerboard analysis generated three-dimensional matrix, where each microtiter plate well contained unique combinations of all three antibacterials as described elsewhere (Hemaiswarya et al., 2008). This modified checkerboard approach employed multiple microtiter plates (6–8) with 50 μL of TQ at concentrations (4–0.125X MIC). The first antibacterial was serially diluted across the plate columns (8–0.125X MIC) using 25 μL volumes, while the second antibacterial was serially diluted undergo row-wise (25 μL volumes with the same concentration range). A total of 100 μL (10^5^ bacterial density suspension) was added with final concentrations representing half the added concentrations due to the subsequent 1:1 dilution during inoculation. The experiments were run in triplicate including control wells without TQ. The fractional inhibitory concentration index (FICI) was computed via summing the FIC values for both antibacterials. The FIC for each antibacterial was calculated by dividing its MIC value in combination by its individual MIC value. Specifically, FICI ≤0.5 signifies synergism, FICI range between >0.5 and ≤1.0 shows an additive effect, FICI value range between >1 and ≤4 defines indifferent effect and FIC > 4 indicates antagonism.

### Quantitative real-time PCR analysis of efflux and cell wall associated genes expression

Total RNA was isolated from identified *S. aureus* strains, which had been exposed to representative antibacterials and/or TQ to analyze the expression of *norA* and cell wall synthesis genes (*PBP2a* and *PBP4*). RNA extraction was performed using Qiazol reagent (Qiagen, Germany) following the manufacturer’s protocol. To eliminate any genomic DNA contamination, the RNA was extracted after treatment with DNAase using Applied Biosystems reagent (Thermo Fisher, Waltham, MA, United States). RNA quality was assessed, with acceptable 260/280 ratio values ranging from 1.8 to 2.0, using a Nanodrop UV spectrophotometer (Thermo Fisher, Waltham, MA, United States). Samples exhibiting values outside this range were eliminated. Complementary DNA (cDNA) synthesis was performed using a high-capacity cDNA reverse transcriptase kit (Applied Biosystems, United States), using 500 ng of total RNA from each sample and following the supplier’s guidelines. In brief, the reverse transcription reaction was done in a 20 µL reaction volume containing 10 µL of the total RNA containing 500 ng, 1 µL of MultiScribeTM Reverse Transcriptase, 0.2 µL of 10X RT random primers, 0.8 µL of 25X dNTP Mix (100 mM), 2 µL of 10X RT Buffer, 1 µL of RNase inhibitor, and 3.2 µL of RNase-free water. The synthesized reaction was done in a 96-well gradient PCR system (Biometra, Life-Sciences, Germany) with a temperature of 25 °C for 10 min, followed by 37 °C for 120 min and then 85 °C for 5 min. The gene expression study was performed using the RotorGene Q-5 Plex real-time PCR system (Qiagen, Germany) in a 20 µL reaction volume using primers, supplied by Thermo Fisher, United States, encoding target genes (Oheagbulem et al., 2023) and QuantiNova SYBER green master mix (Qiagen, Germany) according to the supplier’s instructions with a cyclic condition of initial denaturation at 95 °C for 10 min, 40 cycles of denaturation at 95 °C for 10 s, annealing at 60 °C for 15 s and elongation at 72 °C for 15 s followed by a melting curve analysis (Khamis et al., 2022). The primer sequences for target genes alongside average cycle threshold values are presented in Supplementary Table S1. The fold change was calculated using the 2^−ΔΔCT^ method previously described (Livak and Schmittgen, 2001). The expression of target genes was normalized using *16S rRNA* gene, with the following primer sequence; forward, 5′-GGC​AAG​CGT​TAT​CCG​GAA​TT-3′ and reverse, 5′-GTT​TCC​AAT​GAC​CCT​CCA​CG-3′ (Vaudaux et al., 2002) for its stable expression across different treatments. This was assayed using the GeNorm tool https://genorm.cmgg.be/.

### Molecular docking of TQ with PBP2a and PBP4

To perform molecular docking simulations of selected TQ legends into the active binding sites of PBP2a and PBP4 proteins, the AutoDock Vina software was used (Siddiqui et al., 2022). The crystal structures of PBP2a and PBP4 were downloaded from the Protein Data Bank at https://www.rcsb.org/. The macromolecule preparation was done using Auto Dock Tools 4.2, which included the removal of water, the deletion of the heteroatom and the addition of polar hydrogen. The TQ ligand 3D structure was obtained from PubChem: https://pubchem.ncbi.nlm.nih.gov/. Root Mean Square Deviation (RMSD) as an analytical metric in molecular dynamics simulations was conducted across multiple modes and binding affinities and RMSD values were analyzed to assess pose reliability. RMSD values below 2 Å were considered indicative of stable and relevant binding conformations. The docking process was performed using autodock Vina and Vina Split software. The results were visualized using the Biovia Discovery Studio 2024 client.

### Statistical analysis

The data were analyzed using SPSS version 26 (IBM Corp, Armonk, NY, United States). The Chi-square test was used to analyze categorical data, including the differences in the antibacterial resistance patterns of the recovered isolates from various sources. Stacked bar plots, where sub-columns are calculated as a part of the total column, were used for visualization of the resistance to various antibacterial agents and classes of *S. aureus* isolates using the ggplot (Wickham et al., 2007) package in R software version 4.3.3 (https://www.r-project.org/). Additionally, one-way ANOVA and Tukey’s *post hoc* test were used to evaluate the efficacy of thymoquinone (TQ)/dual antibacterial combination or single antibacterial treatments on the relative expression (2^−ΔΔCT^) analysis of *norA, PBP2a*, and *PBP4* genes in *S. aureus* isolates. The normality and homogeneity among the treatment groups were determined utilizing Shapiro–Wilk’s and Levene’s tests, respectively. All experimental procedures were done in triplicate, and the results were expressed as mean ± standard error of the mean (SEM). The *p*-values were considered statistically significant if they were less than 0.05. Graphs were generated by GraphPad Prism version 8 (San Diego, CA, United States), and R-software version 4.4.3 (R Core Team R, 2024) using pheatmap (Kolde, 2010), ggplot (Wickham et al., 2007), and factoextra (Kassambara and Mundt, 2020) packages.

## Results

### Susceptibility of *S. aureus* to antibacterials

**FIGURE 1:**
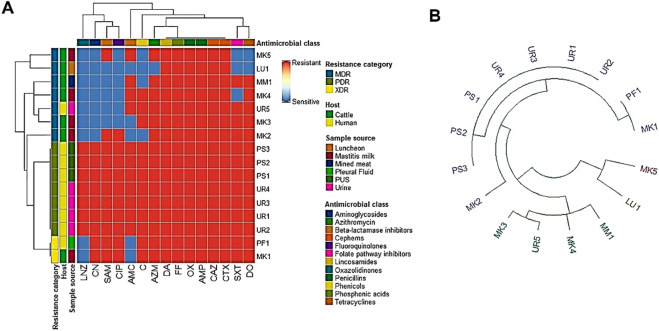
Hierarchical clustering (HA) heatmap **(A)**, and HA dendrogram **(B)** revealing the overall distribution of the tested *Staphylococcus aureus* isolates based on the phenotypic antibacterial resistance pattern. Distinct hosts, sample origins, resistance profiles and antibacterial categories are represented by color codes on the right of the heatmap. UR, human urine; PS, human pus; PF, human pleural fluid; MK, mastitis milk; MM, minced meat; Lu, luncheon; SAM, ampicillin/sulbactam; AMC, amoxicillin/clavulanic acid; SXT, trimethoprim/sulfamethoxazole, C, chloramphenicol; DO, doxycycline; CN, gentamicin; AZM, azithromycin; CIP, ciprofloxacin; LNZ, linezolid; DA, clindamycin; FF, fosfomycin; OX, oxacillin; AMP, ampicillin; CTX, cefotaxime and CAZ, ceftazidime.

**FIGURE 2:**
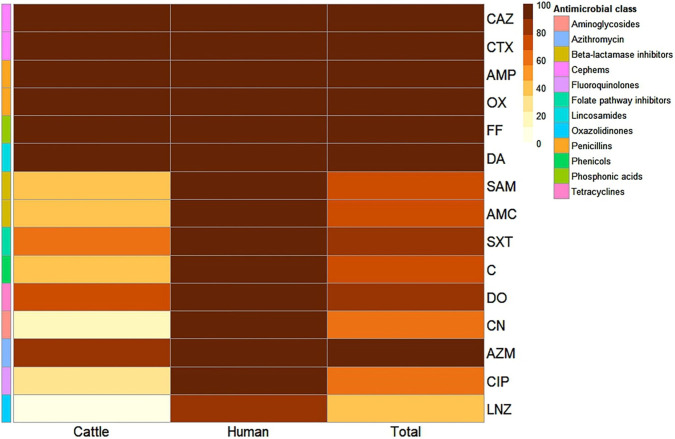
Heatmap illustrating antibacterial resistance profiles of *Staphylococcus aureus* isolates obtained from various sources. The color scale on the right side of the heatmap indicates the percentage of resistance to each tested antibacterial agent. Different antibacterial classes are distinguished using specific color codes. SAM, ampicillin/sulbactam; AMC, amoxicillin/clavulanic acid; SXT, trimethoprim/sulfamethoxazole; C, chloramphenicol; DO, doxycycline; CN, gentamicin; AZM, azithromycin; CIP, ciprofloxacin; LNZ, linezolid; DA, clindamycin; FF, fosfomycin; OX, oxacillin; AMP, ampicillin; CTX, cefotaxime and CAZ, ceftazidime.

Sixteen *S. aureus* isolates were recovered from various sources including urine samples (5), pus specimens (3), pleural fluid (1), cows’ milk (5), minced meat (1) and luncheon (1). These isolates underwent antibacterial susceptibility testing using disc diffusion method against 15 selected antibacterial agents (Figure 1). Universal resistance was observed across all isolates to five antibiotics; clindamycin, fosfomycin, oxacillin, ampicillin, cefotaxime and ceftazidime. The resistance profiles revealed seven PDR strains (designated as UR 1-4 and PS 1-3) and 8 multi-drug-resistant ones (Table 1; Figure 2). *S. aureus* isolates derived from food sources did not exhibit PDR phenomenon and demonstrated high resistance rates to azithromycin, doxycycline, trimethoprime/sulphamethoxazole and amoxicillin/clavulanic acid. Human-derived *S. aureus* strains harbored more resistance determinants compared to food-chain ones (Figures 1, 2). Statistical analysis showed significant differences in the antibacterial resistance patterns of tested *S. aureus* isolates from human, and animal sources to chloramphenicol, gentamicin, ciprofloxacin, and linezolid antibacterials (*p* = 0.019, 0.009, 0.035, and 0.003, respectively). The seven PDR *S. aureus* isolates additionally demonstrated resistance to NS oil and TQ and they underwent disc replacement testing to assess changes in antibacterial susceptibility when exposed to single or combined antibiotic therapies alongside NS oil (Table 2) or TQ (Table 3).

**TABLE 1:** Antibiogram of *S. aureus* isolates from different sources.

Strain ID	Source	Antibacterial agent (inhibition zone diameter, mm)								
		SAM	AMC	SXT	C	DO	CN	AZM	CIP	LNZ
UR1	Urine	R (5)	R (5)	R (9)	R (8)	R (3)	R (10)	R (10)	R (10)	R (10)
UR2	Urine	R (6)	R (5)	R (10)	R (6)	R (4)	R (9)	R (9)	R (9)	R (10)
UR3	Urine	R (8)	R (9)	R (8)	R (5)	R (5)	R (8)	R (8)	R (5)	R (10)
UR4	Urine	R (7)	R (8)	R (8)	R (5)	R (4)	R (8)	R (7)	R (8)	R (10)
UR5	Urine	S (26)	R (5)	R (5)	R (4)	R (9)	S (20)	R (7)	S (23)	S (26)
PS1	PUS	R (9)	R (5)	R (5)	R (10)	R (8)	R (7)	R (6)	R (9)	R (9)
PS2	PUS	R (5)	R (8)	R (5)	R (8)	R (6)	R (8)	R (5)	R (8)	R (8)
PS3	PUS	R (5)	R (6)	R (5)	R (9)	R (6)	R (7)	R (9)	R (7)	R (7)
PF1	Pleural fluid	R (6)	S (26)	R (4)	R (8)	R (5)	R (8)	R (6)	R (9)	S (28)
MK1	Milk	R (7)	S (24)	R (4)	R (5)	R (5)	R (7)	R (5)	R (8)	S (23)
MK2	Milk	R (7)	S (22)	R (4)	S (19)	R (6)	S (19)	R (4)	R (8)	S (26)
MK3	Milk	S (24)	S (21)	R (3)	R (10)	R (7)	S (15)	R (4)	S (23)	S (24)
MK4	Milk	S (17)	R (7)	S (22)	R (9)	R (8)	S (18)	R (3)	S (30)	S (37)
MK5	Milk	R (7)	R (8)	S (35)	S (22)	S (25)	S (20)	R (2)	S (29)	S (25)
MM1	Mined meat	S (16)	R (9)	R (4)	S (27)	R (10)	S (18)	R (4)	S (23)	S (24)
LU1	Luncheon	S (15)	S (22)	S (36)	S (25)	S (26)	S (15)	S (8)	S (24)	S (23)

UR, urine; PS, pus; PF, pleural fluid; MK, milk from mastitic cows; MM, minced meat; Lu, lunchun; R, resistant; S, sensitive; SAM, ampicillin/sulbactam; AMC, amoxicillin/clavulanic acid; SXT, trimethoprim/sulfamethoxazole; C, chloramphenicol; DO, doxycycline; CN, gentamicin; AZM, azithromycin; CIP, ciprofloxacin; LNZ, linezolid. All isolates were resistant to clindamycin, fosfomycin, oxacillin, ampicillin, cefotaxime and ceftazidime.

**TABLE 2:** Modulation of *Staphylococcus aureus* susceptibility to single and combined antibacterials in the presence of *N. sativa* purified oil.

Strain ID	Susceptibility modulation of antibacterials in the presence of *N. sativa* purified oil				
	Single antibacterial		Combined antibacterials		
	Still resistant	Restoration of activity	Still resistant	Synergism	Hinder antagonism
UR2	CZ, CN, OX, FF, LNZ, AZM, DA, AMP, CIP, SXT	CTX^IS^	CZ/OX	CTX/FF, CIP/AZM, CIP/DA	CIP/CN
UR3	CIP, CN, AMC	(CTX, FF)^IS^	Nil	CTX/FF	CIP/AMC
UR1	CTX, FF, CIP, CN, DA, LNZ, AMC, SXT, OX	CZ^IS,^ DO^S^	CZ/OX	(CIP/DA) ^SCV^	Nil
PS2	CIP, LNZ, AMC, CZ	Nil	Nil	(CIP/DA) ^SCV^	CIP/CN
UR5	CTX, LNZ, CZ, AMP, CIP, SXT, AMC, CN	(AZM, DA)^IS^ DO^S^	AZM/DA	CIP/AZM, (AZM/FF, AZM/SXT)^SCV^	Nil
PS1	CN, LNZ, FF	CTX^SCV,^ (SXT, OX, CIP, DO, DA)^S^	AZM/DA	CIP/AMC (CIP/DA) ^SCV^	Nil
UR4	OX, DA, SXT, FF CIP, AMC	(CIP, DO)^S^	Nil	CIP/DA	Nil

UR, urine; PS, pus; AMP, ampicillin; AMC, amoxicillin/clavulanic acid; SXT, trimethoprime/sulphamethoxazole; CIP, ciprofloxacin; DO, doxycycline; LNZ, linezolid; AZM, azithromycin; FF, fosfomycin; OX, oxacillin; DA, clindamycin; CN, gentamicin; CTX, cefotaxime; CZ, ceftazidime; S, sensitive; IS, intermediate sensitive, SCV, small colony variant. All interpretation criteria were based on Clinical and Laboratory Standards Institute guidelines.

**TABLE 3:** Checkerboard data analysis of thymoquinone combination with antibacterials.

Strain	Broth microdilution		Checkerboard data analysis			
	TQ MIC (µg/mL)	MIC (µg/mL) of the drug alone	Drug MIC(µg/mL) at combination with TQ	MIC fold drop at combination		FICI of antibacterial
				TQ	Antibacterial	
UR2	600	CIP (12), AZM (125)	CIP (0.002)/AZM (0.005)	9	CIP (12)/AZM (14)	0.0002
		DA (125), CN (15)	CIP (0.005)/DA (0.002)		CIP (14)/DA (12)	0.0004
		CTX (125)	CIP (0.08)/CN (0.02)		CIP (7)/CN (9)	0.008
			CTX (125)		0.0	1.0
UR3	600	CIP (6), AMC (30)	CIP (0.003)/AMC (0.005)	9	CIP (10)/AMC (12)	0.0006
PS2	300	CIP (12), CN (8)	CIP (0.002)/CN (0.001)	8	CIP (12)/CN (12)	0.0002
UR5	1,000	CIP (25), AZM (125)	CIP (0.25)/AZM (0.25)	10	CIP (6)/AZM (9)	0.012
PS1	300	CIP (6), AMC (8)	CIP (0.02)/AMC (0.01)	8	CIP (8)/AMC (9)	0.004
		DO (8)	DO (0.01)		DO (9)	0.01
UR4	300	CIP (6), DA (125)	CIP (0.02)/DA (0.002)	8	CIP (8)/DA (12)	0.003

UR, urine; PS, pus; TQ, thymoquinone; MIC, minimum inhibitory concentration; FICI, fractional inhibitory concentration index; AMC, amoxicillin/clavulanic acid; CIP, ciprofloxacin; DO, doxycycline; AZM, azithromycin; DA, clindamycin; CN, gentamicin; CTX, cefotaxime. The fractional inhibitory concentration index (FICI) was computed via summing the FIC, values for both antibacterials. The FIC, for each antibacterial was calculated by dividing its MIC, value in combination by its individual MIC, value. FICI ≤0.5 signifies synergism, FICI, range between > 0.5 and ≤1.0 shows an additive effect, FICI, value range between >1 and ≤4 defines indifferent effect and FIC >4 indicates antagonism.

### Antibacterial susceptibility restoration of *S. aureus* strains treated with *N. sativa* essential oil and individual antibacterials using disc replacement analysis

*Nigella sativa* oil modulated resistance to single antibacterials against PDR *S. aureus* strains revealing several important therapeutic implications (Table 2). Additive effects for single antibacterials were noticed upon combination with NS oil in most analyzed strains suggesting that the oil enhanced the antibacterial activities without full synergism. This was particularly notable for beta-lactams and fluoroquinolones across multiple strains. True synergistic interactions were relatively rare and strain-specific. For example, *S. aureus* strain encoding UR2 showed synergism with cefotaxime suggesting that synergistic effects may be dependent on specific resistance mechanisms present in individual strains. Notably, the two-drug combinations with NS essential oil generally showed better activity than single agents with several instances of synergism among examined strains. An interesting phenomenon of SCVs was observed in *S. aureus* strains with code numbers of UR1, PS2, UR5 and PS1.

**FIGURE 3:**
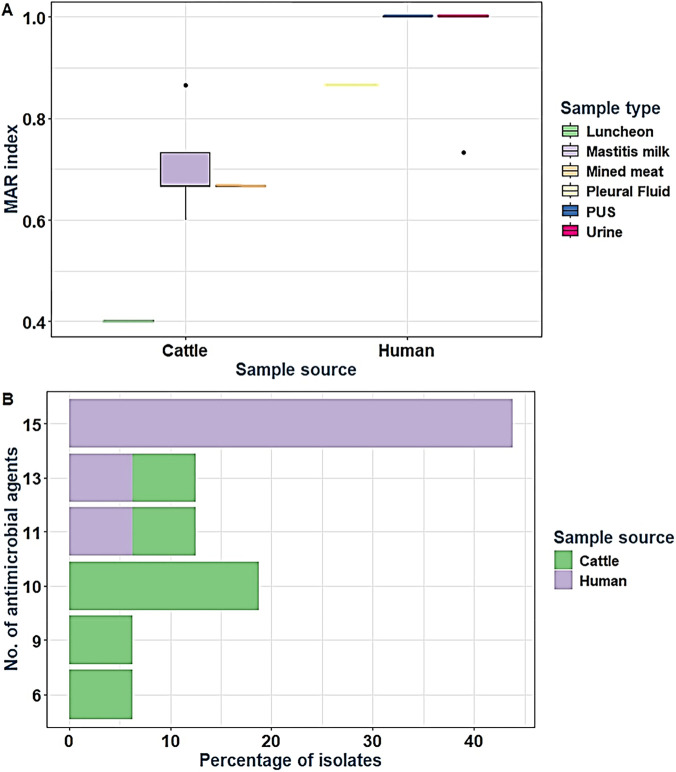
Multiple antibiotic resistance (MAR) index **(A)** and resistance levels to various antibacterial agents **(B)** among tested *Staphylococcus aureus* isolates collected from diverse sources. *In the stacked bar chart **(B)**, frequencies were calculated based on the total number of the examined isolates (n = 16) for each parameter with subcolumns representing proportions relative to the overall column height.

All our tested isolates were resistant to six or more antibacterial agents with MAR index ≥0.4. The majority of *S. aureus* isolates (43.8%) were resistant to all the 15 tested antibacterial agents (Figure 3). There were statistically significant differences in the prevalence of resistance to 15 antibacterial agents and 12 antibacterial classes with MAR index of 1 (*p* = 0.003) among *S. aureus* isolates obtained from human and animal sources.

The investigated phenotypic antibacterial resistance patterns exhibited moderate diversity with five branches and one cluster, including clindamycin, fosfomycin, oxacillin, ampicillin, cefotaxime and ceftazidime (Figure 1A). The examined *S. aureus* isolates exhibited low diversity based on the phenotypic antibacterial resistance profiles. Among the 16 examined isolates, 7 isolates belonged to various lineages. Four branches with two clusters were observed in our results, and one human, and one human isolates (code no. PF1, MK1) were clustered together (Figure 1B).

### Antibacterial susceptibility restoration of *S. aureus* strains treated with *N. sativa* essential oil and two antibacterials using disc replacement analysis

**FIGURE 4:**
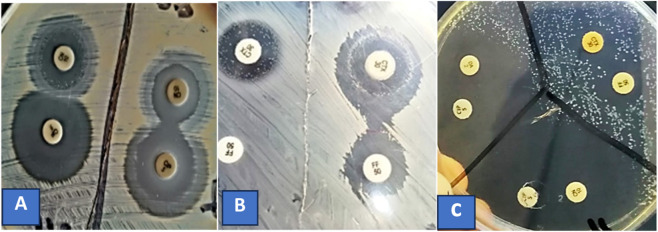
Restoration of antibacterial susceptibility in resistant *Staphylococcus aureus* strains using *Nigella sativa* (NS) oil **(A,B)** and thymoquinone **(C)** combinations therapies as evaluated through disc replacement assays. Panel A (left) demonstrates antagonistic interaction between ciprofloxacin and gentamicin (D-shaped zone around CIP indicating diminished inhibition where antibiotics intersect); combining these antibiotics with NS oil (right) reversed the antagonism in *S. aureus* strain ID UR3. Panel B displays an additive interaction (left) between cefotaxime and fosfomycin; pairing these drugs with NS oil (right) produced synergistic effects against *S. aureus* strain ID UR2. Panel C exhibits synergistic activity when ciprofloxacin was paired with clindamycin or gentamicin in the presence of thymoquinone; meanwhile, fosfomycin and cefotaxime showed an additive interaction. CIP, ciprofloxacin; CN, gentamycin; FF, fosfomycin; CTX, cefotaxime; DA, clindamycin.

*Nigella sativa* essential oil modified resistance to dual antibacterials against PDR *S. aureus* strains demonstrating several vital therapeutic inferences (Table 2). *N. sativa* oil demonstrated additive interactions with specific dual antibacterial combinations. The combination of ceftazidime and oxacillin (CZ/OX) showed additive effects in two strains (Code numbers UR2 and UR1). Additionally, the pairing of azithromycin and clindamycin (AZM/DA) exhibited additive interactions in two strains encoding UR5 and PS1. Other tested strains showed no additive effects with dual antibacterial combinations suggesting that additive interactions are relatively uncommon when NS oil was combined with paired antibacterials. Significant synergistic interactions were observed between NS oil and various dual antibacterial combinations. The most commonly observed synergistic combination was ciprofloxacin with clindamycin (CIP/DA) showing synergistic effects in five strains (Code numbers UR2, UR1, PS2, PS1, and UR4) with two strains exhibiting increased susceptibility and three involving SCVs formation. The appearance of SCVs with these combinations is noteworthy as these variants often have altered metabolism and may represent a bacterial stress response that could potentially lead to persistent infections. The combination of cefotaxime and fosfomycin (CTX/FF) exhibited synergistic effects in two strains (Code numbers UR2 and UR3) both showing sensitive responses (Figure 4). Ciprofloxacin paired with azithromycin (CIP/AZM) demonstrated synergistic activities in two strains designated UR2 and UR5. Additional synergistic combinations included ciprofloxacin with amoxicillin/clavulanic acid (CIP/AMC) in one strain encoding PS1 and azithromycin with both fosfomycin (AZM/FF) and trimethoprime/sulphamethoxazole (AZM/SXT) showing SCVs formation in one strain designing UR5. The combination of ciprofloxacin and gentamycin (CIP/CN) showed antagonism reversal in two strains (Code numbers UR2 and PS2). Additionally, ciprofloxacin paired with amoxicillin/clavulanic acid (CIP/AMC) exhibited antagonism reversal in one strain (UR3). These findings suggest that NS essential oil could overcome antagonistic interactions between certain antibacterial pairs potentially restoring or enhancing their combined antibacterial efficacy in specific *S. aureus* strains.

### Antibacterial susceptibility restoration of *S. aureus* strains treated with thymoquinone and two antibacterials using disc replacement analysis

Thymoquinone exhibited distinct synergistic properties when paired with dual antibacterial compounds on MHA via disc replacement methodology (Figure 4). Synergistic effects were predominantly observed when ciprofloxacin was combined with either clindamycin or gentamicin in the presence of TQ with no SCVs detected around the antibacterial discs. In contrast, combinations involving fosfomycin and cefotaxime exhibited additive interactions.

### Interpretation of modified checkerboard results against pandrug-resistant *S. aureus*

Checkerboard assay data encompassed the MICs of TQ and antibacterials both individually and in combination (Table 3). Thymoquinone was evaluated alongside single antibacterial agent as well as with dual antibacterial combinations with ciprofloxacin being one component of the paired antibacterials.

Both TQ and individual antibacterials exhibited MIC values indicative of bacterial resistance. However, when TQ was combined with ciprofloxacin and one of azithromycin, clindamycin, gentamicin or amoxicillin/clavulanic acid, a significant pharmacodynamic enhancement was observed resulting in 8 to 10-fold reductions in TQ MICs. The antibacterial activity of all tested combinations was restored as reflected by FICI values ranging from 0.0002 to 0.01. These combinations also mitigated potential antagonistic interactions among certain antibacterials. The combination of doxycycline with TQ enhanced antibacterial activity showing strong synergy with FICI of 0.01. However, combining cefotaxime with TQ produced no synergistic effect with FICI of 1.0 indicating no interaction.

### Gene expression profiling of *norA*, *PBP2a*, and *PBP4* in pandrug-resistant *S. aureus* following thymoquinone combination therapy

**FIGURE 5:**
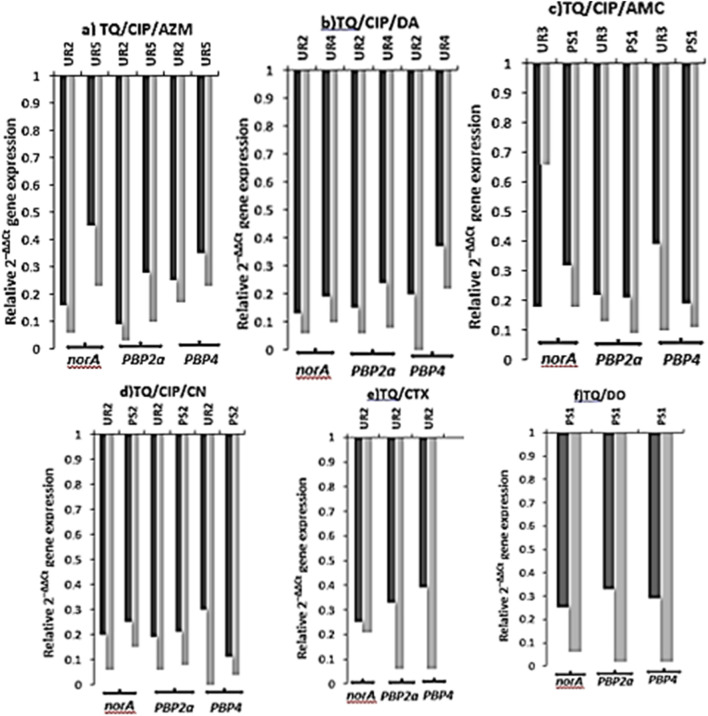
Relative expression (2^−ΔΔCT^) analysis of *norA*, *PBP2a* and *PBP4* genes in six *S. aureus* isolates (ID UR2, UR3, UR4, UR5, PS1 and PS2) following treatment with thymoquinone (TQ)/dual antibacterial combination **(a–d)** or single antibacterial **(e,f)** therapies. Dark bars represent gene suppression under combination treatment protocols, while light bars indicate gene suppression in positive control groups receiving TQ at 0.5XMIC. UR, urine; PS, pus; CIP, ciprofloxacin; AZM, azithromycin; DA, clindamycin; AMC, amoxicillin/clavulanic acid; CN, gentamicin; CTX, cefotaxime; DO, doxycycline.

**FIGURE 6:**
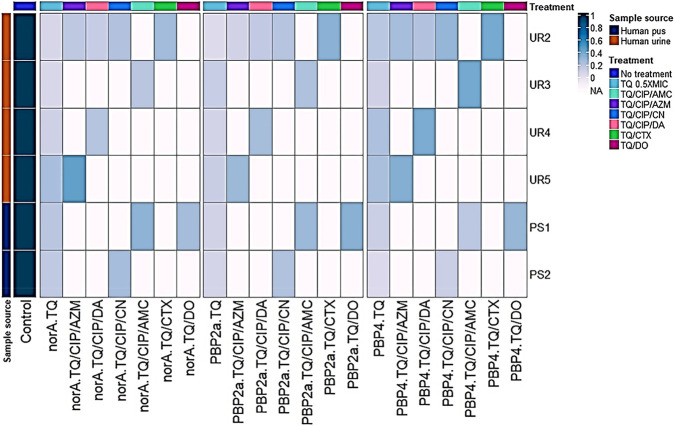
Hierarchical clustering heatmap showing the fold changes in the expressions of *norA*, *PBP2a* and *PBP4* genes among six *S. aureus* isolates following treatment with o.5XMIC thymoquinone (TQ)/dual antibacterial combination or single antibacterial therapies. The scale on the right of the heatmap represents the fold changes of the examined gene in comparison with the transcription levels of the control untreated isolates, which were assigned a value of 1. NA, non-applicable; UR, urine; PS, pus; CIP, ciprofloxacin; AZM, azithromycin; DA, clindamycin; AMC, amoxicillin/clavulanic acid; CN, gentamicin; CTX, cefotaxime; DO, doxycycline.

Expression analysis was conducted on three critical genes (*norA*, *PBP2a* and *PBP4*) in six *S. aureus* strains treated with a combination of TQ, CIP and AMC, DA, AZM or CN or TQ with CTX or DO versus positive controls treated solely with TQ at 0.5XMIC (Figures 5, 6). Substantial gene downregulation was observed for all three target genes across every treatment regimen tested. Gene expression levels were uniformly suppressed below 1.0 relative to untreated controls with values spanning 0.03 to 0.66 throughout all experimental conditions. The triple combination therapies demonstrated more uniform gene suppression patterns when compared to TQ monotherapy controls (0.5XMIC). Strain-specific responses were evident among the six *S. aureus* isolates encoding UR2, UR3, PS1, UR5, PS2 and UR4 with individual strains exhibiting variable levels of gene suppression. The dual combination of DO and TQ demonstrated molecular level suppression of *norA* and *PBP* genes consistent with that of triple combinations (TQ, CIP and AMC, DA, AZM or CN). The reduced expression of *norA* gene indicated the strong efflux pump inhibitory activity of the antibacterials used in each combination therapy. Reduced expression of *PBP2a* and *PBP4* genes in each combination treatment indicated substantial disruption of bacterial cell wall integrity. Although strain designing UR2 demonstrated molecular level suppression of *norA* and *PBP* genes following CTX and TQ treatment (Figures 5, 6), this isolate maintained phenotypic resistance when assessed through disc substitution assays on MHA. The synergistic effects observed in checkerboard assays directly correlated with the degree of *norA*, *PBP2a* and *PBP4* gene downregulation across the tested combinations. There were statistically significant differences (*p* < 0.0001) in the transcriptional modulation of *norA*, *PBP2a,* and *PBP4* genes among thymoquinone (TQ)/dual antibacterial combination or single antibacterial treated and control untreated *S. aureus* isolates (Figure 5). The most significant (*p* < 0.0001) downregulation of *norA*, *PBP2a,* and *PBP4* genes was recorded among all TQ 0.5XMIC-treated *S. aureus* isolates, unlike the control untreated ones (Figures 5, 6).

### Docking analysis of thymoquinone against PBPs

**FIGURE 7:**
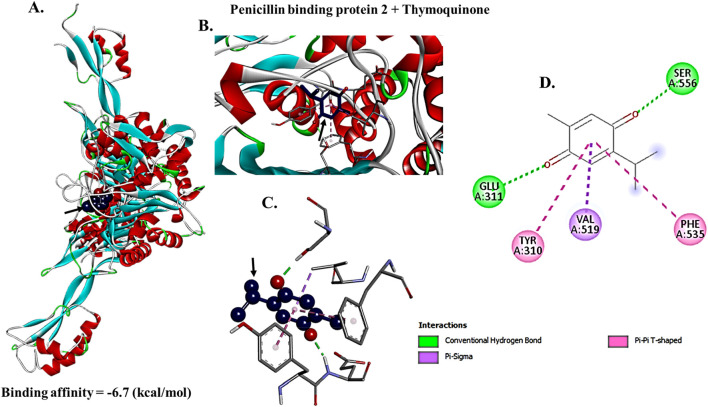
Crystal structure of penicillin binding protein 2 (PBP2a) interactions with thymoquinone. Thymoquinone bound to PBP2a with a binding affinity score of −6.7 Kcal/moL. Docking diagram of thymoquinone combined with PBP2 is shown as a stick model **(A,B)**. Thymoquinone combined with PBP2a at 5 binding site **(C,D)**, distance from best mode (rmsd I. b. = 0.000).

**FIGURE 8:**
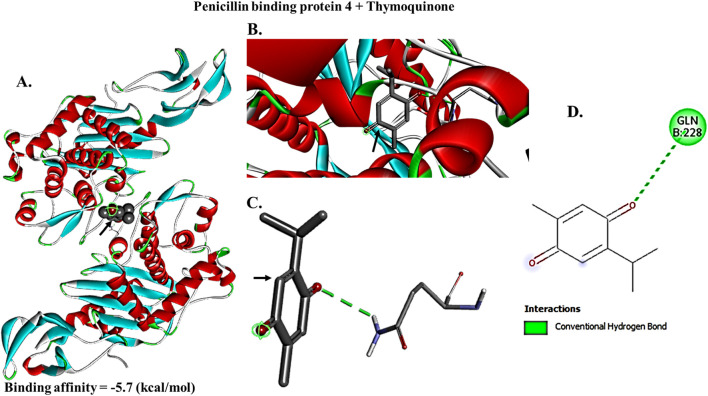
Crystal structure of penicillin binding protein 4 (PBP4) interactions with thymoquinone. Thymoquinone bound to PBP4 with a binding affinity score of −5.7 Kcal/moL. Docking diagram of thymoquinone combined with PBP4 is shown as a stick model **(A,B)**. Thymoquinone combined with PBP4 at one binding site **(C,D)**, distance from best mode (rmsd I. b. = 0.000).

**FIGURE 9:**
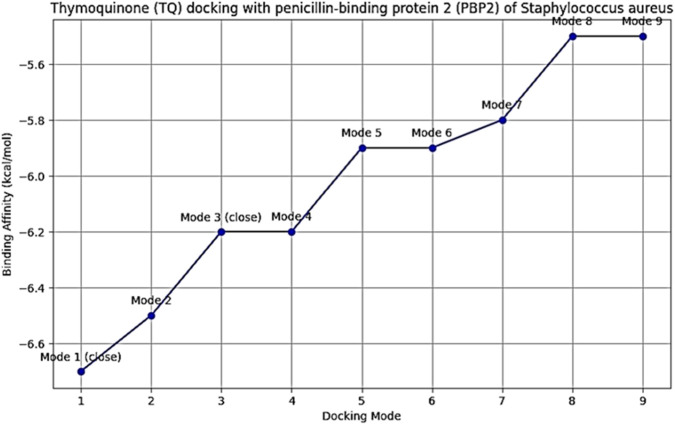
Binding affinities of thymoquinone (TQ) with penicillin binding protein (PBP2) of *Staphylococcus aureus*. The y-axis: binding affinity (Kcal/mol). This axis represents the strength of interaction between TQ and PBP2. More negative values indicate stronger binding. The x-axis: Root Mean Square Deviation (RMSD, Å). This axis shows the deviation of each docking mode from the reference pose. Lower RMSD values suggest a pose closer to the optimal binding configuration. Modes labeled “close” (e.g., modes 1 and 3) indicate poses with low RMSD values suggesting proximity to the optimal binding site and higher reliability.

**FIGURE 10:**
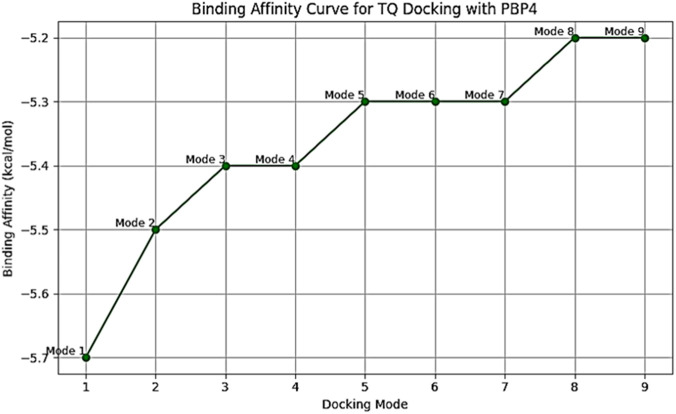
Binding affinity curve for thymoquinone docking with penicillin-binding proteins (PBP4) of *Staphylococcus aureus*. Mode 1: strongest binding affinity at −5.7 kcal/mol indicating the most stable and favorable interaction between TQ and PBP4. Modes 2–3: slightly weaker binding affinities (−5.5 to −5.4 kcal/mol) still considered good and structurally similar to Mode 1. Mode 4: same affinity as mode 3 (−5.4 kcal/mol), but likely represents a structurally different pose. Modes 5–7: binding affinity drops to −5.3 kcal/mol suggesting moderate interaction strength. Modes 8–9: lowest affinity values in the set (−5.2 kcal/mol) indicating the least favorable docking poses among the modes analyzed.

To examine TQ active component direct inhibitory function on PBPs necessary for bacterial cell wall production, TQ ligand interactions with PBP2a and PBP4 protein macromolecules were studied *in silico* using a structure-based virtual screening approach. The molecular docking interactions between TQ active component and PBP2a revealed that TQ interacted with PBP2a with a binding affinity score of −6.7 Kcal/moL via conventional hydrogen bonds at GLU (A:311) and SER (A:556), pi-pi T-shaped interactions at TYR (A:310) and PHE (A:535) and pi-sigma bonds at VAL (A:519) (Figure 7). To examine TQ direct inhibitory function on PBP4, a macromolecule-ligand interaction was established using molecular docking. The results demonstrated that TQ inhibited PBP4 activity with an affinity binding score of −5.7 kcal/moL via a conventional hydrogen bond at GLN (B:228) (Figure 8). The low RMSD (typically <2 Å) showed that the TQ ligand maintained a consistent binding pose suggesting strong and specific interactions with PBP2a and PBP4 (Tables 4, 5; Figures 9, 10). Conversely, high RMSD values may reflect weak or non-specific binding potentially rendering the ligand ineffective. A comparative analysis of TQ docking with penicillin-binding proteins; PBP2a and PBP4 revealed that TQ exhibited stronger binding affinity towards PBP2a. It displayed multiple docking modes with high affinity (ranging from −6.5 to −6.1 kcal/mol) indicating consistent and favorable interactions. In contrast, PBP4 showed moderate binding affinities between −5.5 and −5.2 kcal/mol across its mode. The best docking mode for PBP2a showed a binding affinity of −6.7 kcal/mol, while PBP4’s best mode was −5.7 kcal/mol. This suggests that TQ may act as a more potent inhibitor of PBP2a. These findings imply that while TQ interacts with both proteins, its inhibitory potential is more pronounced against PBP2a making it a promising candidate for targeting this protein in therapeutic applications against *S. aureus*.

**TABLE 4:** Docking modes of thymoquinone with PBP2a based on binding affinity and RMSD values.

Mode	Binding affinity (kcal/mol)	RMSD l.b. (Å)	RMSD u.b. (Å)	Significance of RMSD bounds
1	−6.7	0.0	0.0	Identical to best pose, highly reliable
2	−6.5	61.787	63.549	Very high RMSD, likely irrelevant binding
3	−6.2	1.101	2.652	Low RMSD, similar to best pose
4	−6.2	61.881	63.608	Very high RMSD, likely irrelevant binding
5	−5.9	71.941	73.616	Extremely high RMSD, unreliable pose
6	−5.9	16.474	18.615	High RMSD, weak and distant binding
7	−5.8	15.956	17.128	High RMSD, weak and distant binding
8	−5.5	71.51	72.713	Extremely high RMSD, unreliable pose
9	−5.5	71.864	73.482	Extremely high RMSD, unreliable pose

RMSD, root mean square deviation.

**TABLE 5:** Docking modes of thymoquinone with PBP4 based on binding affinity and RMSD values.

Mode	Binding affinity (kcal/mol)	RMSD l.b. (Å)	RMSD u.b. (Å)	Significance of RMSD bounds
1	−5.7	0.000	0.000	Similar to best pose, reliable binding
2	−5.5	2.012	4.209	Moderate deviation, possibly relevant
3	−5.4	1.220	4.577	Similar to best pose, reliable binding
4	−5.4	14.054	15.715	High deviation, likely irrelevant
5	−5.3	2.182	2.472	Moderate deviation, possibly relevant
6	−5.3	37.693	38.737	High deviation, likely irrelevant
7	−5.3	1.704	3.343	Similar to best pose, reliable binding
8	−5.2	2.260	4.621	Moderate deviation, possibly relevant
9	−5.2	1.621	2.531	Similar to best pose, reliable binding

RMSD, root mean square deviation.

## Discussion

*Staphylococcus aureus* develops resistance mechanisms against all antibacterials, particularly those used for the treatment of infections caused by Gram-positive bacterial species. Antibiotics lost effectiveness due to bacterial natural selection, as reported over six decades ago, leading to the development of extensively drug-resistant and PDR bacterial strains (Ammar et al., 2022; Ammar et al., 2021; Ammar A. M. et al., 2016; Elfaky et al., 2022; Abd El-Hamid et al., 2023; Marwa and Bendary, 2015). Combined antibiotics therapy is the only option for eliminating these bugs and clinical medical practitioners should consider using combined antibacterials even if the bacterial cause is sensitive to a single drug. Extensive clinical usage of combined antibacterials developed resistance to these combinations (Bauernfeind and Petermuller, 1984) and ciprofloxacin was the most common among these combinations (Hailer, 1987). Current combination antibacterial strategies for combating *S. aureus* infections encounters several challenges with uncertain causes including drug interactions that enhance effectiveness, restricted spectrum activity against specific strains, ongoing debates about their efficacy and the emergence of resistance to multi-drug approaches. Consequently, there is an urgent need to improve and minimize the constraints associated with using these combined antibacterial therapies. Although TQ has been extensively studied for its potent *in vitro* bactericidal activity against *S. aureus*, its therapeutic use remains limited due to pharmacokinetic challenges most notably, the C-max being significantly lower (10–30 times) than the MICs required for clinical isolates.

Previous attempts to enhance TQ bioavailability including nanoparticles’ formulations have yielded only modest improvements in C-max. This limitation continues to hinder the approval of *N. sativa* as a clinically viable antibacterial agent against systemic *S. aureus* infections. In our study, we aimed to overcome this barrier by identifying antibacterial agents that synergize with TQ enabling its bactericidal effect at concentrations within the achievable C-max range. We employed phenotypic assays (disc replacement and modified checkerboard) to assess the synergistic interactions and further investigated the molecular mechanisms through quantitative real-time PCR analysis of *norA, PBP2a* and *PBP4* genes regulation in phenotypically drug-resistant strains. For the first time, we also explored the direct inhibitory effects of TQ on PBP2a and PBP4 using molecular dynamic simulations. Pharmacodynamic synergy between TQ and certain dual antibacterial combinations demonstrated (i) restoration of bactericidal activity without the emergence of SCVs and (ii) downregulation of *norA, PBP2a* and *PBP4* genes. Molecular dynamics simulations further confirmed that TQ ligands directly inhibited PBP2a and PBP4 proteins.

Owing to the broad range of benefits of *N. sativa* pharmacological actions, sales of black cumin-containing products in United States increased by 200% in 2017 (Tyler et al., 2018). Several clinical studies and trials have been attempted to explore the safety of standardized *N. sativa* seed oils and TQ at different dosages. Rats supplemented with *N. sativa* up to 1.0 g/kg revealed no toxicity effects on liver functions (Dollah et al., 2013). In another study, a dose of 5 mg of TQ per kg of body weight in rats and less than 50 mg of TQ per adult per day in humans were safe according to the Organization for Economic Co-operation and Development guidelines (Ramalingam et al., 2021).

In this study, certain PDR *S. aureus* strains demonstrated prominent susceptibility owing to the strong antibacterial activity of TQ/CIP and TQ/DO dual treatment regimens. However, several strains remained unresponsive to antibacterial effects of these binary combinations. The enhanced efficacy of triple-agents’ treatment (TQ/CIP with AMC, DA, AZM or CN) against these previously non-susceptible *S. aureus* isolates can be explained by concurrent synergistic mechanisms including i) TQ’s dual action through efflux pump inhibition (targeting NorA and MepA systems) and cell wall biosynthesis interference (Ahmad et al., 2024)., ii) ciprofloxacin’s interference with bacterial DNA replication via DNA gyrase and topoisomerase IV inhibition and iii) complementary antibacterial targets provided by these agents creating multi-pathway bacterial inhibition. The antibacterial combinations incorporating CIP with supplementary agents in conjunction with TQ exhibited potent bacterial suppression, which stands in stark contrast to previous researches those documented limitations in quinolone-based combinatorial treatments due to i) variable synergistic responses observed among bacterial isolates from different geographical locations (Bauernfeind and Petermuller, 1984), ii) significant debate surrounding ciprofloxacin combination therapy with conflicting studies reporting both synergistic and antagonistic interactions (Hackbarth et al., 1986; Kaatz et al., 1989) and iii) synergistic effects may be restricted to MSSA isolates while showing limited efficacy against MRSA ones (Rohner et al., 1989).

The bacterial isolates in the current study demonstrated *in vitro* TQ resistance with elevated MIC values of 300–1,000 μg/mL. Previous investigations on *S. aureus* strains reported TQ-MIC ranges of 8–256 μg/mL (Kouidhi et al., 2011). Such elevated TQ-MIC levels precluded TQ monotherapy for clinical *S. aureus* infections based on i) impractical pharmacokinetic parameters, particularly regarding achievable TQ plasma peak concentrations (C-max) and ii) suboptimal TQ absorption characteristics and limited bioavailability. Earlier researches showed that TQ-encapsulated nanostructured lipid carriers reached plasma C-max levels of 3,342 ± 224.76 ng/mL in rats following 20 mg/kg oral administration, while conventional TQ suspension (20 mg/kg) achieved only 1160.5 ± 270.85 ng/mL plasma C-max (Elmowafy et al., 2016; Ansar, 2022). TQ exhibits delayed absorption kinetics with 58% bioavailability and 23 min lag time (Alkharfy et al., 2011). Nevertheless, the current study revealed that low TQ concentration (2 μg/mL) achieved 8- to-10- fold decrease in MIC values surpassing those previously reported with essential NS oil (Mouwakeh et al., 2019) suggesting feasibility of attaining therapeutic blood concentrations (C-max) and validating the clinical potential of these combination therapies for systemic *S. aureus* infections. These findings demonstrated positive agreement with the observed downregulation of *norA, PBP2a* and *PBP4* genes. Dual antibacterial combinations with TQ such as TQ/DO as well as triple combinations involving TQ and ciprofloxacin alongside agents like AMC, DA, AZM or CN resulted in significant downregulation of *norA*, *PBP2a* and *PBP4* genes compared to control conditions.

Although significant suppression of *norA*, *PBP2a* and *PBP4* genes was observed with TQ/CTX combination therapy, the tested isolate exhibited resistance with the development of SCVs upon TQ/CTX exposure indicating an indifferent antibacterial response. This sustained resistance may be attributed to selective PBP2a/PBP4 downregulation without concurrent suppression of PBP1/PBP3 enabling the remaining functional PBPs to preserve resistance mechanisms through CTX preferential binding characteristics (Łeski and Tomasz, 2005). Suppression of *norA*, *PBP2a* and *PBP4* genes observed following TQ/CTX combination therapy might be due to alternative resistance mechanisms, compensatory gene expression and post-transcriptional regulation. Moreover, TQ/CTX/FF achieved bactericidal effects through comprehensive cell wall targeting; FF blocks early peptidoglycan synthesis (MurA), CTX disrupts late synthesis (PBPs), while TQ enhances penetration and suppresses resistance mechanisms creating inescapable cell wall disruption (Łeski and Tomasz, 2005; Chan et al., 2016).

In the present work, TQ demonstrates direct PBP inhibition evidenced through molecular docking with strong binding affinities; PBP2a (−6.7 kcal/moL) and PBP4 (−5.7 kcal/moL). PBP2a binding involves five hydrogen bonds’ sites (GLU A:311, SER A:556, TYR A:310, PHE A:535 and VAL A:519) located outside the penicillin binding domain effectively bypassing resistance mutations (E→K237 and V→E470) found in highly β-lactam resistant *S. aureus* (Katayama et al., 2004). This direct binding approach disrupts cell wall cross-linking and creates osmotic vulnerability. PBP4 binding at GLN B:228 in the non-penicillin domain complements PBP2a inhibition and affects ceftizoxime resistance. The dual-target strategy provides comprehensive cell wall disruption through direct protein inhibition at alternative binding sites explaining TQ effectiveness against PDR by circumventing traditional resistance mechanisms (Łeski and Tomasz, 2005; Ahmad et al., 2025; Fani et al., 2013). There were no direct studies demonstrating specifically TQ binding to or inhibiting PBP2a. In the present work, TQ exhibited stronger binding affinity towards PBP2a; meanwhile, PBP4 showed moderate binding affinities. Most research on TQ’s antibacterial mechanisms focused on ATP synthase inhibition (Ahmad et al., 2015), membrane disruption, reactive oxygen species generation (Goel, 2018) and downregulation of virulence and toxin genes. Some studies explored TQ’s synergistic effects with antibiotics against MRSA, which involved PBP2a indirectly. For example, Alsufyan et al., 2025 investigated TQ’s activity against MRSA and MSSA and targeted *mecA* gene encoding PBP2a, but did not confirm direct inhibition. Similarly, Dera et al., 2021 reported enhanced antibacterial effects of TQ in combination with antibiotics suggesting indirect interactions with resistance mechanisms.

## Conclusion

Certain *S. aureus* strains were susceptible to the bactericidal effects of TQ at MICs below 3.0 μg/mL aligning with the serum C-max pharmacokinetic parameter. However, strains with higher MICs were unlikely to be effectively treated systemically limiting TQ’s clinical utility. This limitation may stem from TQ’s poor absorption and low bioavailability. The use of safe plant extracts alongside dual antibacterials is considered acceptable as combining antibacterials is a common practice in treating bacterial infections. When TQ is paired with conventional dual antibacterials, it enhanced the sensitivity of *S. aureus* to bactericidal effects even at low concentrations and helped to restore their efficacy. Pharmacodynamic studies showed downregulation of *norA*, *PBP2a* and *PBP4* genes. Molecular dynamics simulations further confirmed that TQ directly inhibited PBP2a *and* PBP4 through TQ ligand binding. The results indicated that the applied combinations helped in overcoming the limitations associated with TQ clinically use. Moreover, TQ was evidenced to be an effective adjunctive therapy that enhanced the efficacy of conventional antibacterials against resistant *S. aureus* making it a suitable option for treating systemic infections in clinical settings.

## Data Availability

The original contributions presented in the study are included in the article/Supplementary Material, further inquiries can be directed to the corresponding authors.
