# Supplementary material for: Thymoquinone combined with conventional antibiotics against pandrug-resistant Staphylococcus aureus: a pharmacodynamic and molecular simulation strategy to overcome bioavailability limitations
Source: Front Pharmacol. 2026 Mar 12;17:1735325. doi: 10.3389/fphar.2026.1735325 (PMC13018160; doi:10.3389/fphar.2026.1735325)
Supplement: Supplementary file 1 [file Supplementaryfile1.docx]

**Supplementary Table 1.** Oligonucleotide primers used for detection of *norA, PBP2* and *PBP4* genes among *S. aureus* isolates

| Target gene | Primer sequence (5ˋ-3ˋ) | CT average | Reference |
| --- | --- | --- | --- |
| *16S rRNA* | F: GGCAAGCGTTATCCGGAATT  R: GTTTCCAATGACCCTCCACG | 16.97- 22.67 | **Pourmand *et al.,* 2014** |
| *norA* | F: TTGCTATTACGGGTGGCGGT  R: TCAATCCGCCTGCAAAGCCT | 19.56- 28.52 | **Young *et al.,* 2020** |
| *PBP2* | F: CAACTAATGAAACAGAAAGTCGTAA  R: TAATGTATGTGCGATTGTATTGCTA | 19.83- 30.81 | **Oheagbulem *et al.,* 2023** |
| *PBP4* | F: ACGATGTTTTACCAAGTGATTTTAG  R: CCAATGATAGTGAATAATGGATGT | 20.01- 30.18 |  |

*norA*: multidrug resistant efflux pump encoding gene, PBP: penicillin binding protein, CT: cycle threshold

**Pourmand, M.R. *et al.* (2014)** Evaluation of expression of NorA efflux pump in ciprofloxacin resistant staphylococcus aureus against hexahydroquinoline derivative by real-time PCR’, *Acta Medica Iranica*, 52(6), pp. 424–429.

**Young, M.; Walsh, D.J.; Masters, E.; Gondil, V.S.; Laskey, E.; Klaczko, M.; Awad, H.; McGrath, J.; Schwarz, E.M.; Melander, C.; et al. (2020)**: Identification of *Staphylococcus aureus Penicillin* Binding Protein 4 (PBP4) Inhibitors. Antibiotics*.*11, 1351.

**Oheagbulem, A.S., Oche, D.A., Akuakolam, and Olajide Akinnibosun, O. (2023)**: Detection of PBP2a and PVL genes among *Staphylococcus aureus* and their methicillin-resistant strains isolated from a hospital in Sokoto Town. Microbes and Infectious Diseases. 4(4): 1210-1218.
